# Superplastic Deformation of Al–Cu Alloys after Grain Refinement by Extrusion Combined with Reversible Torsion

**DOI:** 10.3390/ma13245803

**Published:** 2020-12-18

**Authors:** Kinga Rodak, Dariusz Kuc, Tomasz Mikuszewski

**Affiliations:** Faculty of Materials Engineering, Silesian University of Technology, Krasińskiego 8, 40-019 Katowice, Poland; dariusz.kuc@polsl.pl (D.K.); tomasz.mikuszewski@polsl.pl (T.M.)

**Keywords:** KoBo extrusion, mechanical properties, Al–Cu alloys, microstructure and superplastic deformation

## Abstract

The binary as-cast Al–Cu alloys Al-5%Cu, Al-25%Cu, and Al-33%Cu (in wt %), composed of the intermetallic θ-Al_2_Cu and α-Al phases, were prepared from pure components and were subsequently severely plastically deformed by extrusion combined with reversible torsion (KoBo) to refinement of α-Al and Al_2_Cu phases. The extrusion combined with reversible torsion was carried out using extrusion coefficients of λ = 30 and λ = 98. KoBo applied to the Al–Cu alloys with different initial structures (differences in fraction and phase size) allowed us to obtain for alloys (Al-25%Cu and Al-33%Cu), with higher value of intermetallic phase, large elongations in the range of 830–1100% after tensile tests at the temperature of 400 °C with the strain rate of 10^−4^ s^−1^. The value of elongation depended on extrusion coefficient and increase, with λ increasing as a result of α-Al and Al_2_Cu phase refinement to about 200–400 nm. Deformation at the temperature of 300 °C, independently of the extrusion coefficient (λ), did not ensure superplastic properties of the analyzed alloys. A microstructural study showed that the mechanism of grain boundary sliding was responsible for superplastic deformation.

## 1. Introduction

Superplasticity is a diffusion-controlled process that refers to the ability of a metallic material to demonstrate elongation by more than several hundred percent without visible necking under tensile tests at relatively high temperature (0.5–0.7) Tm (where Tm is the absolute melting temperature) and low strain rate [1,2,3]. Superplastic deformation is mainly controlled by three mechanisms that operate during high temperature deformation: grain boundary sliding, dislocation slip/creep, and diffusion creep or directional diffusion flow [2,4]. From this, superplastic deformation may be accompanied by various processes, for instance, grain boundaries migration; static/dynamic grain growth; and grain rotation, recovery, and recrystallization [1,3]. Moreover, superplasticity is a characteristic feature of materials with stable, equiaxed, and fine (less than 10 μm) grains. Suerplasticity process covers various metallic materials, one of which is Al alloys due to their low density and high specific strength. For this reason, they are widely utilized in the aerospace and automobile industries. In practice, these alloys have particles of secondary intermetallic phases that restrict resistance to deformation. The superplastic properties may improve formability of alloys with reduced deformation resistance [3,5].

Some superplastic alloys are already industrially produced and used in practice. Among the most known and useful alloys are the Al–Cu–Mg alloys, with elongation of more than 600% [6], and Al–3Li–0.5Zr alloy, with elongation to failure of 1035% at 370 °C [7]. Highashi et al. [8] experienced exceptional superplastic elongation of 5500% in aluminum bronze at 800 °C and 6.3 × 10^−3^ s^−1^ strain rate. Much research has been reported in superplasticity of metals such as in magnesium-, iron-, titanium-, and nickel-based alloys [1,3,9].

Alloys with superplastic properties have eutectoidal or eutectic composition, although they have not been applied in practice due to unsuitable mechanical properties, although they possess good plasticity. In these alloys, the plasticity of one phase is different from the other due to the difference in crystal structure and/or chemical composition, i.e., the two phases are not equally easy to deform [10,11,12]. According to the literature, plastic deformation starts in the soft phase while the hard phase could still be in the elastic state [4,12]. As the plastic deformation proceeds further, the soft phase is strain hardened and internal stresses at interfaces drastically build up via dislocation accumulation and pile up [10,13,14]. Thus, there is a stress gradient and a strain gradient, which lead to the build-up of back stress [13,14]. Eventually, co-deformation of the two phases begins. Temperature increase causes a change in deformation mechanisms not only in grain interior but also, mainly, in interfaces. This factor primarily allows deformation of such materials. The second factor is connected with the applied technique of deformation. The high shear strain imposed by severe plastic deformation (SPD) can refine the grains in materials down below the ultra-fine-grained (UFG) regime. The phases in alloys can also be significantly and uniformly distributed, unlike the unprocessed samples. The SPD processing very often may be connected with temperature increase during deformation. This phenomenon of SPD may simplify superplastic deformation. Valiev et al. [15] utilized high-pressure torsion (HPT) to process Al–Cu–Zr alloy and obtained elongation of 250% at a low temperature of 220 °C. This confirmed that the SPD process is an important way to increase plasticity of alloy.

The strengthened Al–Cu alloys are commonly used for structural engineering applications, and in civil engineering [13,16]. In practice, these alloys are used in heat treatment state after supersaturation and aging. As-cast alloys with a higher fraction of intermetallic phases can also be useful in engineering applications due to some interesting properties. The intermetallic phases are particularly responsible for formability, mechanical properties, and fracture behavior of as-cast Al–Cu alloys. Fracture toughness and formability decrease with increased fraction of intermetallic phases [3,14]. Intermetallic phases are responsible for increased mechanical properties, and as a result, the application of these alloys at higher temperatures is possible [11,16].

Extrusion combined with reversible torsion (KoBo) is one of the SPD methods [17]. The complex state of stress and deformation enables deformation of practically non-deformable materials. Under conditions induced by KoBo, when the process is realized with higher deformation intensity, it is possible to obtain higher strength properties. Additionally, temperature of deformed material can rise enough to generate some structural changes, indicating the occurrence of dynamic recovery and recrystallization [17,18], and as a consequence, the observed structure becomes composed very often of equiaxed grains, which are typical for increase of plastically properties. 

Thus, in this article, the structural and mechanical properties of grain refinement of Al–Cu alloy with a high-volume fraction of Al_2_Cu phase using the KoBo method is presented. For this reason, alloys that nearly cross the eutectic line, have hypoeutectic composition, and have eutectic composition were analyzed. Method samples refined by KoBo were tensile tested at high temperature to obtain superplastic properties.

## 2. Materials and Methods

The binary Al–Cu alloys with 5 wt % of Cu (Al_5_Cu), with 25 wt % of Cu (Al_25_Cu), and with 33 wt % of Cu (Al_33_Cu), were prepared from pure Cu (99.99 wt %) and Al (99.99 wt %). The alloys were obtained by melting in the Leybold–Heraues furnace. The alloy components were melted using high frequency generator under purified argon atmosphere and cast into a steel mold of cylindrical shape of 50 mm diameter. After cooling, the ingots were removed from the molds, and next were machined to 49.5 mm in diameter and were cut to the length of 100 mm. Then the alloys were extruded using KoBo technique to form rods with diameters of 9 and 6 mm that corresponded to reduction coefficients of λ = 30 and λ = 98, respectively. The coefficient was expressed as follows: λ = [do]^2^/[dk]^2^, where do—initial diameter of ingot, dk—diameter of rod after KoBo. 

The severe plastic deformation tests were carried out with the modernized KoBo 2.5 MN horizontal hydraulic press. The recipient temperature was about 130 °C. All deformation tests were carried out at constant angle of die reverse-rotation +/−8°. The die oscillation frequency was 5 Hz. The tensile tests were performed on a Zwick/Roell 100 machine (Zwick Roell AG, Ulm, Germany) at room temperature, 300 °C, and 400 °C, with a rate of 10^−4^ s^−1^. The dimensions of the samples were diameter (d) = 4 mm, length (lo) = 50 mm. The microstructure was analyzed by the optical microscope Olympus GX71 (Olympus, Glasgow, UK) and the scanning electron microscope (SEM) LEO GEMINI 1525 (Leo Inc., NY, USA) operated at 30 kV. Additionally, a scanning transmission electron microscope (STEM) Hitachi HD-2300 A (Hitachi-Science&Technology, Tokyo, Japan) equipped with a Field Emission Gun (FEG) operated at 200 kV was used for microstructure characterization on longitudinal sections of the extruded billet. For microstructure examination, we used transmitted electron (TE) and Z contrast (ZC) imaging. The metallographic specimens were mechanically grinded and polished, and then were treated by electropolishing in 80% ethanol and 20% perchloric acid solution combined with anodic oxidation in Barker’s reagent. For STEM investigations, foils with a diameter of 3.0 mm after electrolytic thinning were used. 

The X-ray phase analysis was performed on an X’Pert3 Powder diffractometer (Malvern Panalytical, Chester, UK) using a copper anode lamp (λCuK-1.5406 Ǻ) supplied with a current of 30 mA at a voltage of 40 kV. The recording was made in 0.02° steps in the angular range from 10° to 90°2. The tests were carried out on solid samples. The identification of the crystalline phases was performed using the International Centre for Diffraction Data (ICDD PDF-4+) database.

## 3. Results and Discussion

**Figure 1:**
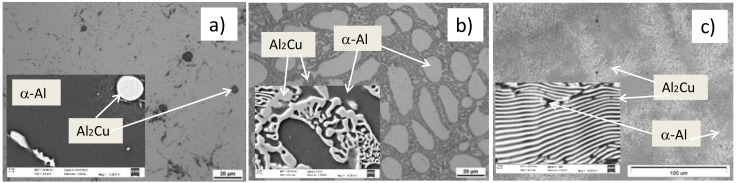
Microstructure of (**a**) Al–Cu alloy with 5 wt % of Cu (Al_5_Cu), (**b**) with 25 wt % of Cu (Al_25_Cu), and (**c**) with 33 wt % of Cu (Al_33_Cu) in initial state.

The microstructures of Al_5_Cu, Al_25_Cu, and Al_33_Cu alloys at as-cast state are presented in Figure 1. The mass weight of Cu in Al_5_Cu alloy was on the limit of maximum Cu solubility in Al. Therefore, the particles of Al_2_Cu phase were visible in dendrites of α phase in a wide range of sizes (large particles with eutectic composition and dispersoids) (Figure 1a). Increase of Cu content led to increase of interdentritic regions in Al_25_Cu alloy. The interdentritic regions consisted of massive eutectoid composition (α + Al_2_Cu phase) and dendrites of α-Al matrix (Figure 1b). Micrometric lamellar structure of the θ-Al_2_Cu and α-Al phase was characteristic for Al_33_Cu alloy (Figure 1c).

**Figure 2:**
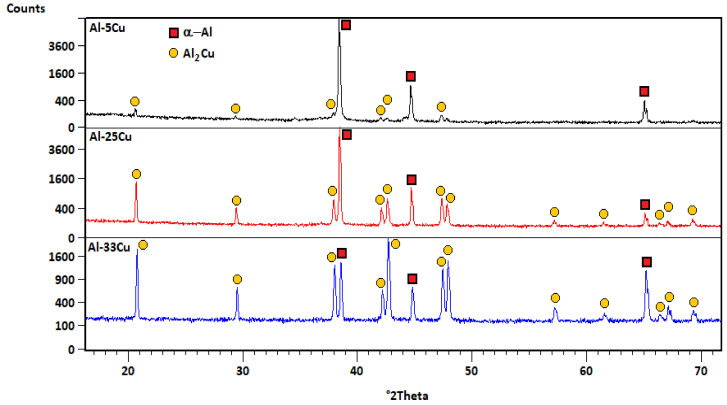
X-ray diffraction patterns of Al–Cu alloys.

The X-ray analysis showed the presence of an aluminum-based solid solution α-Al (face-centered cubic lattice) phase and an intermetallic Al_2_Cu (C16, tetragonal lattice) phase (Figure 2).

**Figure 3:**
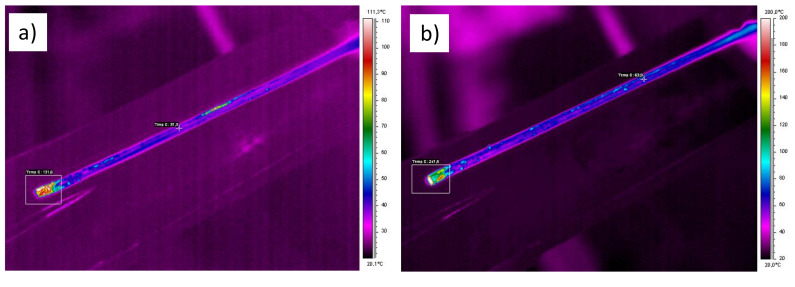
Thermographical pictures of surface samples of Al–Cu alloys after extrusion combined with reversible torsion (KoBo) processing with λ = 98: (**a**) Al_5_Cu alloy, (**b**) Al_25_Cu alloy.

During KoBo tests, we recorded temperature for samples that left the device using the ThermaCam SC640 (Flir Systems Inc., Portland, OR, USA) mobile thermal imaging system equipped with software for thermographic analysis. Examples of thermograms for the Al_5_Cu and Al_25_Cu alloys after using the KoBo method are shown in Figure 3. The temperature was recorded in the range from approximately 37° to 132° for the Al_5_Cu alloy deformed with λ = 98, and in a range from approximately 63° to 247° for the Al_25_Cu alloy deformed with λ = 98. Similar changes in temperature recorded as those for Al_25_Cu alloy were observed for the Al_33_Cu alloy. Differences in recorded temperatures between Al_5_Cu and Al_25_Cu alloys followed from thermal conductivity coefficient of the tested materials. Thermal imaging revealed an increase in temperature on the surface of the material. However, we cannot exclude the potential of a higher increase in temperature inside the sample, where material was intensively deformed.

**Figure 4:**
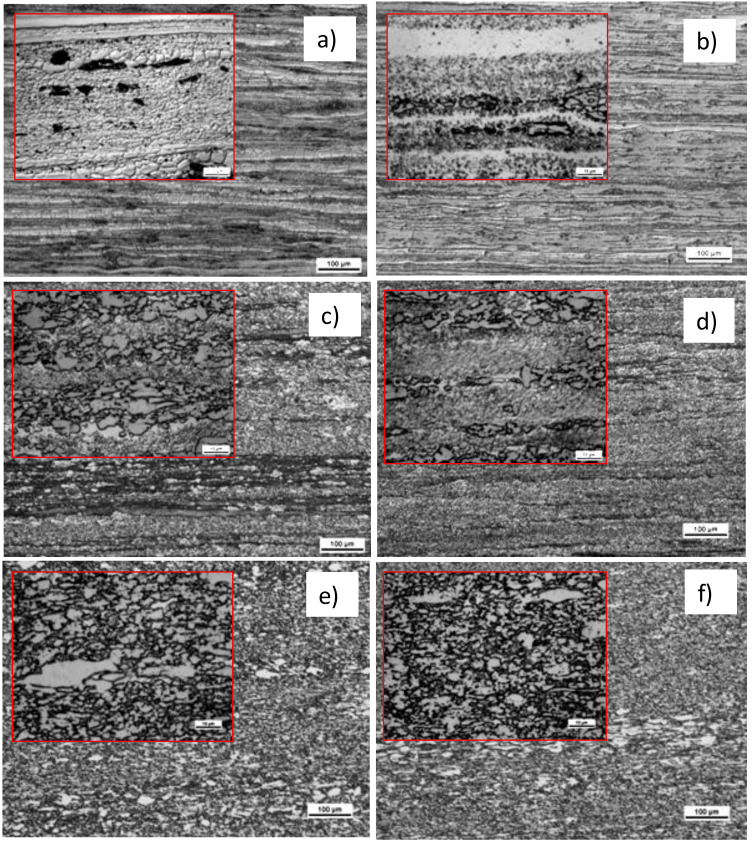
Microstructure of Al–Cu alloys after KoBo deformation: (**a**,**b**) Al_5_Cu alloy, (**c**,**d**) Al_25_Cu alloy, (**e**,**f**) Al_33_Cu alloy, (**a**,**c**,**e**) λ = 30, (**b**,**d**,**f**) λ = 98.

After KoBo deformation, the microstructures visible in the longitudinal section showed characteristic fibrous structure as a result of the extrusion process (Figure 4). Increase in deformation did not introduce noticeable changes in microstructure. Massive regions of eutectoid composition occasionally observed in A_l5_Cu alloy were shown to not be deformed. It was evident that α-Al phase underwent recovery as a result of temperature increase. Visible in Figure 4a are grains of α-Al phase with different grain sizes. The difference in grain sizes may have been the result the localization of fine Al_2_Cu phases that were not uniformly distributed during KoBo. In Figure 4b, the grains of α-Al phase are not visible, meaning that the grains were very small and essentially invisible when using light microscopy (LM) imaging. In Al_25_Cu alloy, the refinement process occurred for θ-Al_2_Cu and α-Al phases (Figure 4c,d). SPD process was connected with distribution of Al_2_Cu phase, which assured a more equiaxial structure compared with sample in as-cast state. Independently of deformation value, heterogeneity in microstructure was visible (Figure 4c,d). The Al_2_Cu phase areas were clearly separated from the α-Al phase areas. Refined Al_2_Cu phase was elongated in the extrusion direction. Although the KoBo process did not introduce homogeneity in the arrangement of the individual θ-Al_2_Cu and α-Al phases for Al_25_Cu alloy, lamellar structure observed for Al_33_Cu alloy promoted homogeneity in microstructure and made the microstructure consist of near equiaxed intermetallic Al_2_Cu and α-Al phases (Figure 4e,f). This was a consequence of eutectic structure, which is the most susceptible to fragmentation and formation of equiaxed grains.

**Figure 5:**
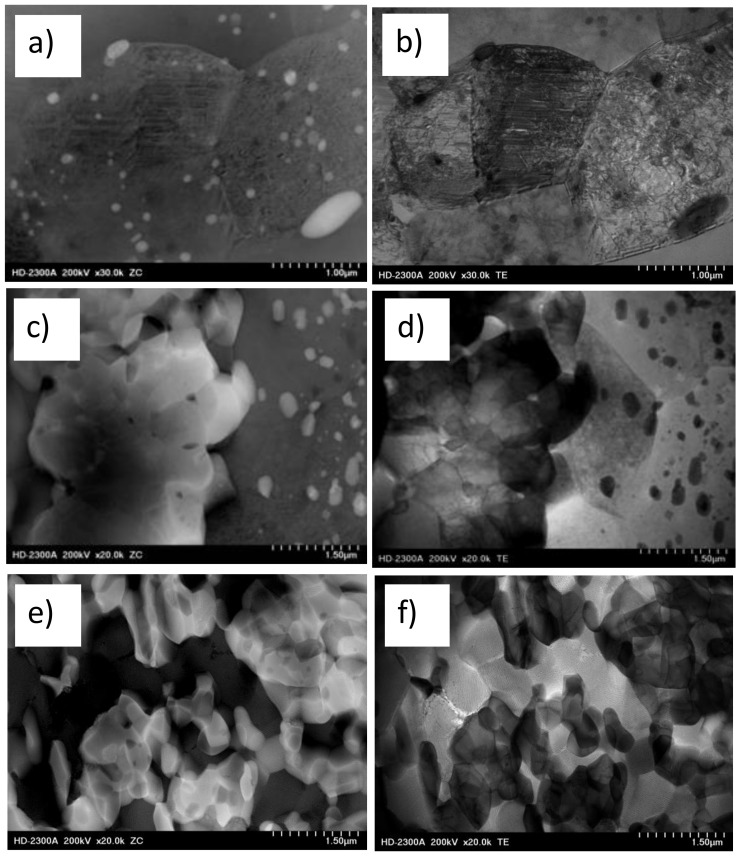
TEM images showing the characteristic microstructures of Al–Cu alloys after KoBo processing at λ = 98. The microstructures on the left side represent Z contrast (ZC) images, while the microstructures visible on the right side represent transmitted electron (TE) images. (**a**,**b**) Al_5_Cu alloy, (**c**,**d**) Al_25_Cu alloy, (**e**,**f**) Al_33_Cu alloy.

Since there were no significant microstructural changes observed using LM, we selected samples after λ = 98 deformation for further STEM investigations in order to focus our attention mainly on the processes accompanying the fragmentation and rearrangement of individual phases. The choice of the deformation with λ = 98 suggested that the structure was more refined and more homogeneous. To show differences between θ-Al_2_Cu and α-Al matrix phases and to show dislocation structure of individual phases, we recorded the microstructures by using ZC imaging (left side of Figure 5) and TE imaging (right side of Figure 5). The bright phases in the left side represent θ-Al_2_Cu phase, which in figures in right sides is shown as dark phases. 

In Al_5_Cu alloys, small Al_2_Cu particles acted as effective barriers of dislocation movement and were responsible for blocking of grain/subgrain boundaries (Figure 5a). The grains of α-Al matrix were visible as equiaxed structures with high dislocation density inside. The diffraction contrast of individual grains means that misorientation between grains was high (Figure 5b). In Al_25_Cu alloy, the role of small and very small particles of Al_2_Cu phase was the same as in Al_5_Cu alloy (Figure 5c,d). Observed massive microareas of Al_2_Cu phase were refined with the characteristic effect connected with creation of grain/subgrain boundaries as a result of dislocation accumulation and their rearrangement (Figure 5d). Even through the microstructure of Al_2_Cu phase was not completely fragmented (Figure 5c), there were observed microvoids pointing to the initiated process of grain refinement (Figure 5c). The microcracks were formed at the boundaries of new grains/grains or at the contact point of three grains. The observed phenomenon proved that first the fragmentation of the structure occurred, as the process of dislocation generation and rearrangement, and then the phase fragmentation process occurred. This process was especially visible when the TE and ZC images were combined, as shown in Figure 5c,d. The generation and rearrangement of dislocations proved the deformability of the intermetallic phase. The examples of generated voids shown in the Figure 5c,d may indicate the initiated process of grain boundary sliding. Temperature increase during KoBo deformation may enable this process. Similar phenomena of creating dislocation boundaries and then phase fragmentation through mutual separation of individual blocks and their mutual rotations were observed in the Al_33_Cu alloy (Figure 5e,f). In this case, the grain refinement process was facilitated by the lamellar structure of the eutectic alloy.

Mechanical properties extruded by KoBo samples are shown in Table 1.

High fraction of α-Al phase in microstructure was characterized by high plasticity. For sample Al_5_Cu, elongation to fracture (Ac) was over 45%, while for Al_33_Cu alloy (Ac), it was about 7%. Ultimate tensile strength (UTS) of Al_5_Cu alloy after the KoBo extrusion reached 160 MPa. Increase of Cu content in alloys (Al_25_Cu and Al_33_Cu alloys) resulted in the increase of strength. Al_25_Cu alloys in terms of UTS were in the range of 280–330 MPa. For Al_33_Cu alloys, the UTS was in range of 450–480 MPa. Strengthening by the intermetallic particles in Al_25_Cu alloy and Al_33_Cu alloy was accompanied by a drastic drop of ductility. For Al_25_Cu alloy, the elongation to fracture (Ac) was about 12%; this value decreased to 7% for Al_33_Cu alloys. Interesting tensile test results were obtained for the alloys deformed with increasing value of deformation. An increase in the λ value caused a slight decrease in UTS with simultaneous increase in elongation. This indicated a favorable phenomenon related to an increase in both strength and plastic properties. The observed high strength properties were a combination of various strengthening mechanisms, i.e., precipitation strengthening, deformation strengthening by generating high dislocation density, and strengthening by grain boundaries (refinement process). Improvement of plastic properties may be attributable to the more homogeneous microstructure with ultra-fine-grained equiaxed grains with high misorientation angles.

The superplastic tensile stress–strain curves at 300 °C and 400 °C are shown in Figure 4. Additionally, the results of mechanical measurement are displayed in Table 2. 

**Figure 6:**
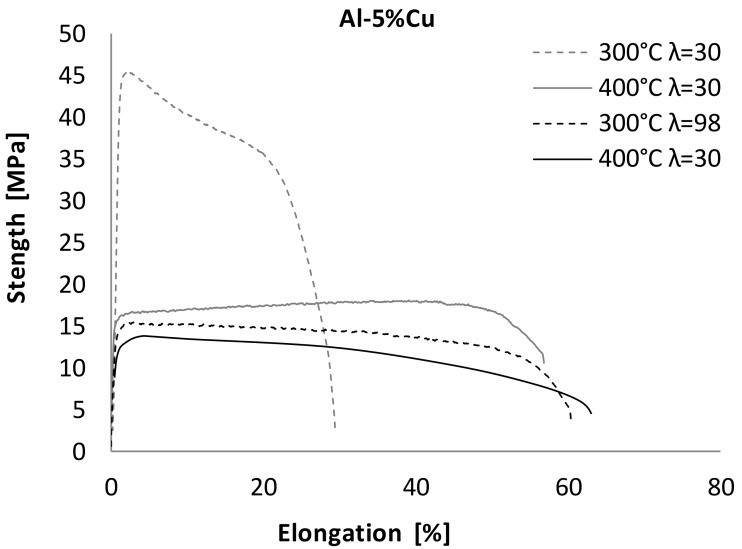
The superplastic tensile stress–strain curves for Al_5_Cu alloy.

**Figure 7:**
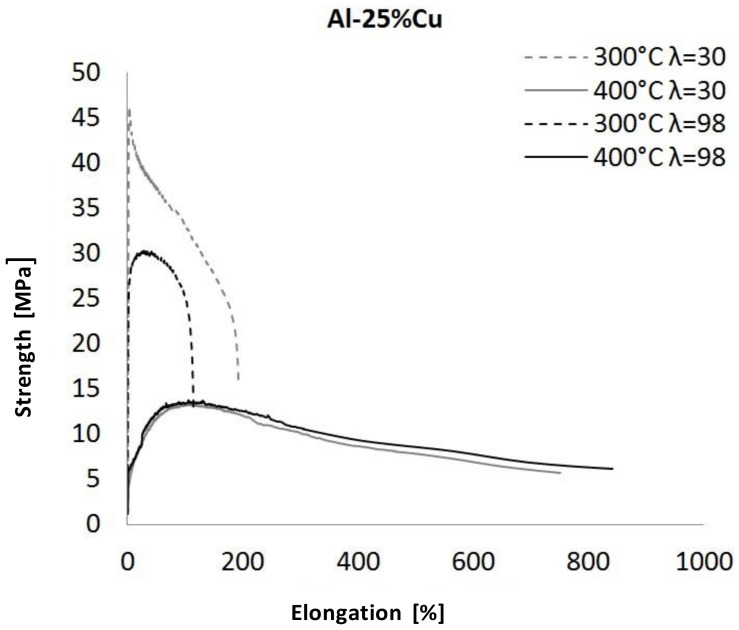
The superplastic tensile stress–strain curves for Al_25_Cu alloy.

**Figure 8:**
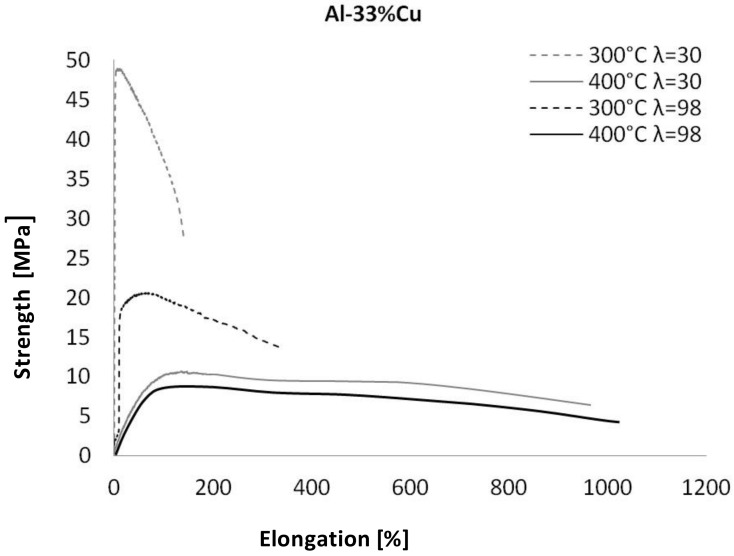
The superplastic tensile stress–strain curves for Al_33_Cu alloy.

From Figure 6, Figure 7 and Figure 8, we see that the flow stress and elongation depended on the deformation temperature and value of λ. As shown in Figure 6, the flow stress curves of Al_5_Cu alloy for 300 °C and λ = 30 were raised sharply to the peak values of about 46MPa and then decreased quite rapidly, with a strain failure of about 30%. As the sample was deformed at λ = 98, the value of peak stress decreased and was about 15MPa. It should be noted that the deformation value was also responsible for the strain increase. The elongation was about 60%. For samples deformed at 400 °C with λ = 30 and λ = 98, the value of peak stress was in range from 14 MPa to 18 MPa. Simultaneously, the samples reached failure in the range from −57% to 63%. It should be noted that samples deformed at 300 °C with λ = 98 were characterized by a similar level of elongation and stress as samples deformed at 400 °C. It was found that the Al_5_Cu alloys had maximal elongation at a temperature of 400 °C. The typical characteristic of flow curves (approaching a steady state) indicated the occurrence of softening effect, which began to be dominated by the acceleration of dynamic recovery during deformation. 

The influence of the deformation temperature on the superplasticity effect in the case of the Al_25_Cu alloy was significant (Figure 7). The superplastic properties of the alloy were evidently demonstrated at the higher deformation temperature. For samples deformed at 400 °C with λ = 30 and λ = 98, the flow peaks were comparable and amounted to approximately 13–14 MPa. Simultaneously, the samples reached failure in the range from 787% to 827%. Generally, the increase in the deformation value in the case of samples deformed at the temperature of 400 °C led only to an increase in elongation. As the samples were deformed at 300 °C, the increase of λ value influence on flow peaks decreased and increased in elongation. However, in this temperature, the alloys did not show evident superplastic properties. 

Similar deformation characteristics as those of the previously described Al_25_Cu alloy were observed for the Al_33_Cu alloy (Figure 8). In this case, an increase in the elongation (about 987% and 1085% for the deformation of λ = 30 and λ = 98, respectively) were noted during deformation at a temperature of 400 °C, with a lowering value of the stress of approximately 10–11 MPa. The samples of Al_33_Cu alloy deformed at 300 °C maintained quite a high level of peak stress equal to 49 MPa and 21 MPa for λ = 30 and λ = 98, respectively. The elongation value did not exceed 350% for samples deformed with λ = 98. On the basis of performed experiments, we must note that the experimental alloys had maximal superplasticity at a temperature of 400 °C. The minimum value of the stress peak and maximum elongation to failure were obtained for Al_33_Cu alloy with the equiaxed ultrafine-scale structure. 

**Figure 9:**
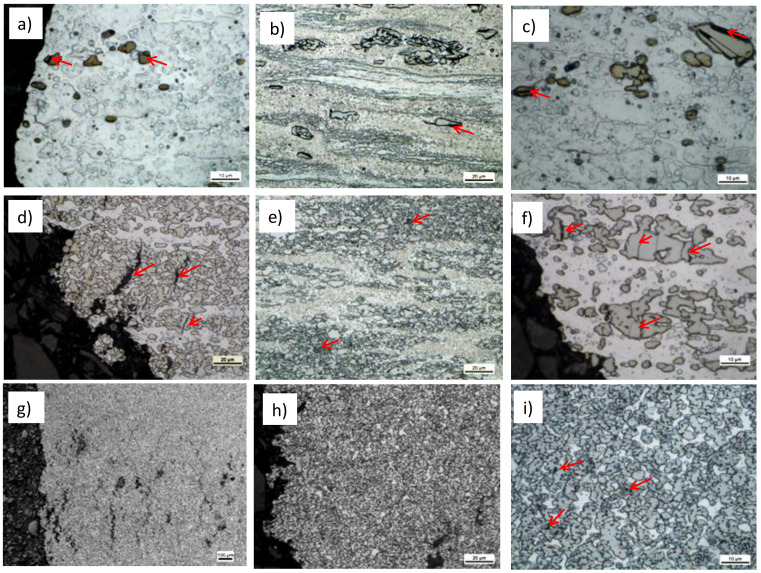
Microstructure of Al–Cu alloys after tensile test at 400 °C: (**a**–**c**) Al_5_Cu alloy, (**d**–**f**) Al_25_Cu alloy, (**g**–**i**) Al_33_Cu alloy.

The microstructures presented in Figure 9 show selected examples of high-temperature deformation of alloys at the temperature of 400 °C for λ = 98. First of all, the microstructural changes visible near the fracture site were taken into account in order to visualize the material fracture mechanism. From Figure 9a–c, it was visible that many of the large- and intermediate-sized particles cracked or detached from the matrix because of their random orientation. Since there were relatively few precipitates in the alloys of high and medium size, the nature of the cracking depended mainly on the matrix, which was plastic and well deformable. Discontinuities visible in interfaces as a result of the mismatch of the crystallographic lattices were much more common than in the process of material decohesion as a result of intermetallic phase cracking. Very small precipitates presented in the matrix (Figure 9a,c) did not support the material cracking process. Small particles rarely fractured, hence reducing crack formation. The fraction of precipitation did not change with deformation increase. The important role of ultra-fine precipitates of Al_2_Cu phase reduced the grain size with deformation. This was important in the process of superplasticity and may have affected increase in elongation. 

In the Al_25_Cu samples, cracks were generated in Al_2_Cu phase and spread in this phase in the direction perpendicular to the tensile direction, as shown in Figure 9d. Moreover, the formation of large discontinuities was favored by numerous cracks present in the massive, non-separated areas of intermetallic phases (Figure 9f). Very often, the span of the crack was determined by the size of the phase. During the next deformation, the cracks merged and a distinct fracture was formed, which was not stopped by the plastic phase. The crack propagated through one intermetallic particle into another, likely due to their proximity (Figure 9e). The fracture was stopped only in the area of the plastic matrix (Figure 9d). 

In the case of the Al_33_Cu alloy, where the distances between the individual phases were much smaller, the developed fracture was retained or absorbed by the α-Al phase. In this case, many cracks were formed (Figure 9g–i). The course of cracks was not as pronounced as in the case of the Al_25_Cu alloy, which was characterized by a heterogeneous structure with an irregular distribution of α-Al and Al_2_Cu phases. The bond between the particle and matrix appeared to be exceptionally strong. Meanwhile, the fine grain structure often increases a material’s ability to tolerate plastic deformation without fracture. 

According to the literature [3] and performed mechanical investigations of superplasticity properties, it should be noted that the effect of deformation temperature on superplasticity is closely related to the microstructure. Due to the rise in plastic deformation temperature, the atomic energy of the alloy is increased, and the activity of atoms is higher [1,2]. Therefore, the binding force of the atoms decreases, resulting in a significant reduction of the dislocation barrier, and thereby reducing the flow stress. On the basis of the obtained data, for microstructural analysis using STEM, we chose samples deformed at the temperatures of 300 °C and 400 °C with λ = 98. This selection was made on the basis of the obtained superplasticity characteristics presented in Figure 6, Figure 7 and Figure 8. Moreover, we considered the fact that a high value of deformation results in a much greater homogeneity of microstructure and in grain refinement [19,20,21]. Therefore, one should expect an increase in superplasticity. 

**Figure 10:**
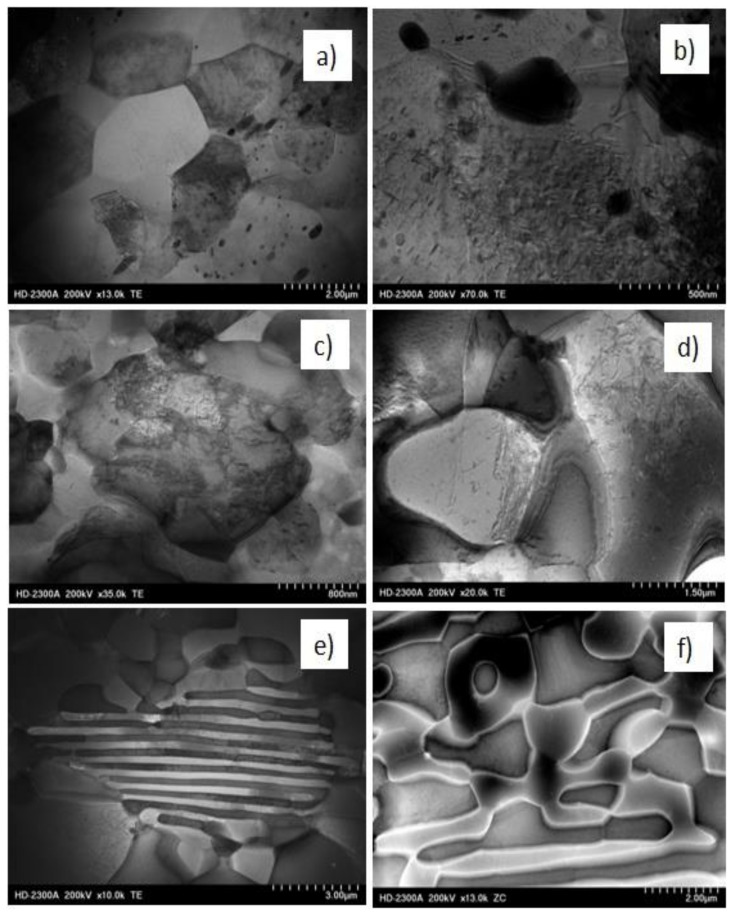
Scanning transmission electron microscope (STEM) images showing the characteristic microstructures of Al–Cu alloys after tensile test at 400 °C. The microstructures on the left side represent ZC images, while the microstructures visible on the right side represent TE images. (**a**,**b**) Al_5_Cu alloy, (**c**,**d**) Al_25_Cu alloy, (**e**,**f**) Al_33_Cu alloy.

**Figure 11:**
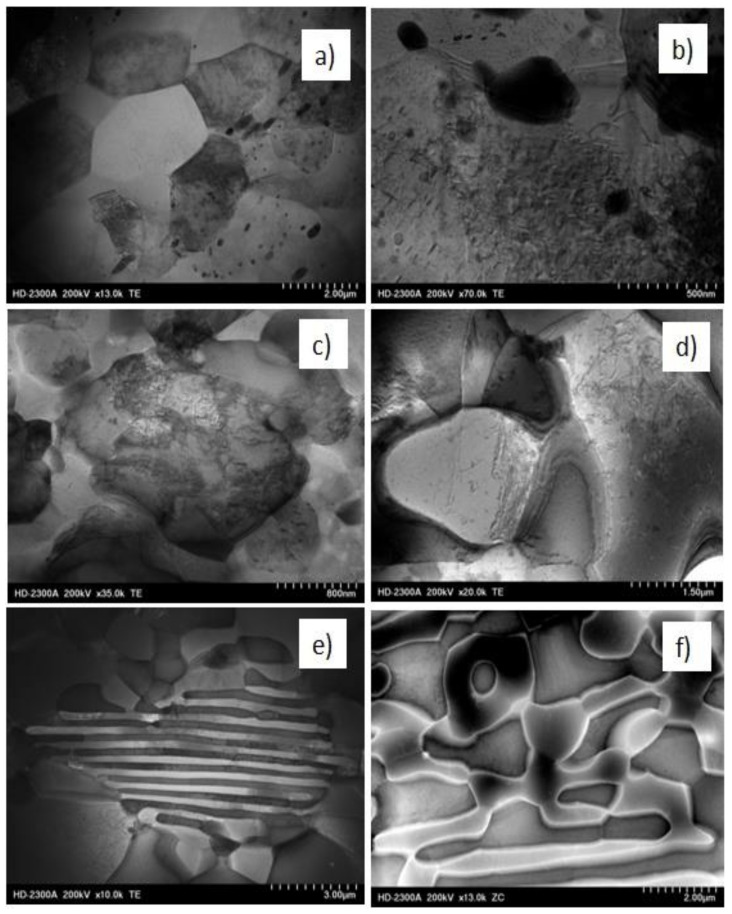
STEM images showing the characteristic microstructures of Al–Cu alloys after tensile test at 400 °C. The microstructures on the left side represent ZC images, while the microstructures visible on the right side represent TE images. (**a**,**b**) Al_5_Cu alloy, (**c**,**d**) Al_25_Cu alloy, (**e**,**f**) Al_33_Cu alloy.

STEM observations (Figure 10) showed the presence of clearly visible α-Al phase grains with high angle boundaries as a result of deformation (Figure 10a,b). In the new grains, a great number of dislocations occurred as a result of deformation at 300 °C. High density of dislocations inside the grains was the result of the presence of a large number of Al_2_Cu phase precipitates. For samples deformed at 400 °C, the dislocation amount was much less (Figure 11a,b). Equiaxial and elongated grains (in the tensile direction) with different sizes were also observed. Figure 10a evidently demonstrates that dislocations were formed in grains during deformation at 300 °C and eventually transforming into subgrains/grains (Figure 10b) with increased temperature. Therefore, the strengthening stage was found to be related to the continuous accumulation of defects and formation and growth of the grains. From these findings, we considered the contribution of intragranular deformation. The structural changes reflect the character of the curves shown in Figure 6. The high density of dislocation in the Al_5_Cu alloy in particular resulted in a greater value of stress peak.

In the Al_25_Cu alloy deformed at the temperature of 300 °C, the dislocations were presented both in the massive phase of Al_2_Cu and α-Al phase (Figure 10c,d). Dislocations present in the Al_2_Cu phase as a result of deformation created numerous dislocation tangles without signs in creation of low- or high-angle boundaries, as were visible for the α-Al phase. This phenomenon indicates differences in the deformation process of both phases. The existence of subgrains and dislocation tangles contributed in a minor way to slide and rotation of Al_2_Cu phase. STEM observations performed for Al_25_Cu alloy deformed at 400 °C showed the presence of well-defined grains/subgrains inside massive Al_2_Cu phase. The microvoids observed in Al_2_Cu phase accelerated the process of grain boundary slipping (Figure 11c). Dislocations accumulated in α-Al phase as a result of deformation generated the new boundaries and multiplication of new grains as a process of continuous recovery (Figure 11d). A probable mechanism of superplasticity is the high angle grain boundary sliding [1,22]. This situation is evidently characteristic for Al_33_Cu alloys. Although the STEM microstructure of the specimens after deformation at 300 °C in some areas was visible as lamellar structure with dislocation density inside Al_2_Cu phase and α-Al phase (Figure 10e,f), we nevertheless found visible examples of equiaxial phases that were fragmented. The evident example of samples with occurrence of grain boundary sliding is shown in Figure 11e, where grains gradually twisted and moved along the tensile direction. When the stress reached its critical value, the alloy was fractured. As presented in Figure 11e, grain boundary sliding occurred more readily for superfine grains with high grain boundaries, an example of which was the eutectic Al_33_Cu alloy. Maximal elongation shown in 400 °C tensile process was the result of the high mobility of grain boundaries (Figure 11e). 

There is only limited information in the literature in terms of the theme connected with understanding of the deformation process of the two-phase microstructure of Al–Cu alloy with a high volume fraction of secondary phase on mechanical properties. For example, in a study by [23], Equal Channel Angular Pressing (ECAP) was successfully applied on a lamellae eutectic alloy of Al_33_Cu at 400 °C, up to an equivalent strain of ≈8. After deformation, a homogeneous fine equiaxed duplex microstructure with an average size of 1.1 mm was obtained. Obtained by using KoBo deformation, microstructures of AlCu alloys are connected with ultrafine near-equiaxed grains of α-Al and Al_2_Cu phase. The obtained grain size and somewhat homogeneous microstructure are capable of achieving superplastic properties. The refinement in the microstructure means that the fraction of grain boundaries, which are responsible for superplastic properties, is increasing, because in the area of grain boundary, the main processes related to superplastic flow take place. After high temperature (400 °C) deformation with λ = 98, the microstructure remained more equiaxed and the microstructure was still fine grained; for this reason, the elongation was higher. The thermally stable and equiaxed grains provided evidence of GBS occurrence. Detailed investigations using scanning transmission electron microscopy showed that grains of the α-Al phase had a higher density of dislocations than Al_2_Cu phase during deformation at 400 °C (Figure 11d,f). This situation was connected with the fact that for Al_2_Cu phase, the processes of recovery can proceed easier than for the α-Al phase. The obtained results are in accordance with the literature [24,25]. During deformation in Al_2_Cu phase, we observed a mechanism connected with non-conservative motion of glide dislocation. This mechanism provided rapid diffusion channels during plastic deformation and indicated structural instability. The high population of vacancies observed in Al_2_Cu alloys facilitated the diffusion of the alloying elements; thus, it may have accelerated de-alloying of Al from Al_2_Cu. In addition, it is conjectured that the easy climb of dislocations driven by dislocation interaction will reduce the glide ability of a dislocation, limiting plastic shear under mechanical loading [25].

On the basis of the obtained results, we found that the intermetallic phase can inhibit the GBS. The nanoscale particles could effectively make the microstructure stable and prevent grain growth so that superior plasticity could be achieved. It is also worth noting that the nanoscale/ultrafine precipitates were of high melting point so as to inhibit the grain growth effectively, with this situation being especially typical for Al_5_Cu and Al_25_Cu alloys.

These mechanical characteristics demonstrated that Al–Cu alloys with high fraction of intermetallic phase are characterized by the typical fine-grained superplasticity.

## 4. Conclusions

1. The KoBo method allowed grain refinement of the AlCu alloys to the micrometric level.
2. Recipient heating and additionally severe plastic deformations contributed to temperature increase; as a result, the processes related to the formation of equiaxial grains with a large fraction of high-angle boundaries were observed. This microstructure is helpful in achieving superplastic properties.
3. The applied value of deformation (λ) played a significant role in the deformation of KoBo. If the samples were deformed with λ = 98, then the grains were refined and the structure was more homogeneous. Deformation increase resulted in an increase of plastic properties with a decrease in strength properties.
4. The samples deformed at 400 °C showed superplastic properties. This problem mainly concerned the Al_25_Cu and Al_33_Cu alloys. Both the phase composition (a large fraction of the intermetallic phase and a large fraction of high angle boundaries) guaranteed superplastic properties of the analyzed alloys.
5. The eutectic Al_33_Cu alloy, due to its specific structure and considerable grain refinement, had the highest elongation during tensile test at high temperature. The Al_25_Cu alloy did not provide comparable value in an elongation to the Al_33_Cu alloy due to the high heterogeneity in the microstructure.
6. The grain boundary sliding mechanism was already initiated during KoBo deformation. During KoBo deformation, microcracks formed, which were observed in the Al_2_Cu phase and in interfaces areas. This microstructural elements in the conditions of superplastic deformation facilitated deformation using the GBS mechanism.

## Figures and Tables

**Table 1:** Measured mechanical parameters: yield strength (YS), ultimate tensile strength (UTS), and elongation to fracture (Ac) of deformed Al–Cu alloys with KoBo processing.

Alloys	Reduction Coefficient	UTS (MPa)	YS (MPa)	Ac (%)
Al_5_Cu	λ = 30	208	75	46
	λ = 98	191	104	55
Al_25_Cu	λ = 30	328	92	12
	λ = 98	280	126	13
Al_33_Cu	λ = 30	446	438	9
	λ = 98	483	398	7

**Table 2:** Measured mechanical parameters: stress peak (σ_max_), elongation to failure (ε_max_) of deformed Al–Cu alloys.

Alloys	Mechanical Properties	300 °C		400 °C	
		λ = 30	λ = 98	λ = 30	λ = 98
Al_5_Cu	σ_max_ (MPa)	46	15	18	14
	ε_max_ (%)	29	60	57	63
Al_25_Cu	σ_max_ (MPa)	46	30	13	14
	ε_max_ (%)	192	114	787	827
Al_33_Cu	σ_max_ (MPa)	49	21	11	10
	ε_max_ (%)	140	345	987	1085
